# Optimizing treatment selection and sequencing decisions for first-line maintenance therapy of newly diagnosed advanced ovarian cancer

**DOI:** 10.1016/j.gore.2022.101028

**Published:** 2022-06-17

**Authors:** Jeffrey C.H. Goh, Charlie Gourley, David S P Tan, Angélica Nogueira-Rodrigues, Hesham Elghazaly, Marc Edy Pierre, Gonzalo Giornelli, Byoung-Gie Kim, Flavia Morales–Vasquez, Alexandra Tyulyandina

**Affiliations:** a Department of Oncology – Cancer Care Services, Level 5, Joyce Tweddell Building, Royal Brisbane & Women’s Hospital, Butterfield Street, Herston, Queensland 4029, University of Queensland, St Lucia, Australia; b Edinburgh Cancer Research UK Centre, MRC IGMM, University of Edinburgh, Western General Hospital, Crewe Road South Edinburgh, EH4 2XR, UK; c Department of Haematology-Oncology, National University Cancer Institute, Singapore; d Federal University of Minas Gerais, DOM Oncologia, Grupo Oncoclınicas, EVA Brazilian Group of Gynecologic Cancer, LACOG, Porto Alegre, Brazil; e Clinical Oncology Head of Medical Research Center MASRI, Ain Shams University, Cairo, Egypt; f Gynecology Oncology Unit, Luis Carlos Samiento Angulo Cancer Treatment and Research Center - CTIC,Bogotá, Colombia; g Department of Oncology, Instituto Alexander Fleming, Buenos Aires, Argentina; h Department of Obstetrics and Gynecology, Samsung Medical Center, Sungkyunkwan University School of Medicine, Seoul, South Korea; i Department of Medical Oncology, Instituto Nacional de Cancerología, Mexico City, Mexico; j Department of Clinical Pharmacology And Chemotherapy, N. N. Blokhin Russian Cancer Research Center, Moscow, Russia

**Keywords:** Ovarian cáncer, PARP inhibitors, Genetic testing, *BRCA* mutations, Olaparib

## Abstract

The incidence and mortality rates of ovarian cancer are increasing globally. Ovarian cancer is diagnosed at an advanced stage in 80% of women. After standard, platinum-based, front-line chemotherapy, poly (ADP-ribose) polymerase (PARP) inhibitors and antiangiogenic agents are successfully employed as maintenance strategies for newly diagnosed, advanced ovarian cancer patients. Landmark clinical studies, including SOLO-1, PAOLA-1, PRIMA, and VELIA, have provided crucial insights on optimizing first-line maintenance treatment using PARP inhibitors. A group of ovarian cancer experts, primarily from low- and middle-income countries, met in September 2019 to discuss new developments for the first-line treatment of ovarian cancer and its implications.

Key implications of the evolving clinical data included: (1) olaparib or niraparib maintenance therapy appears to be the preferred choice for patients with *BRCA*1/2 mutations; hence, *BRCA* testing is beneficial in identifying these patients; (2) niraparib monotherapy and olaparib in combination with bevacizumab have demonstrated significant benefit in progression-free survival (PFS) in homologous recombination deficiency (HRD)-positive patients; (3) bevacizumab, niraparib alone, or observation can be an alternative for HRD-negative patients; (4) further data is warranted to explore the role of PARP inhibitors in treating HRD-negative, ovarian cancer patients to confirm findings of the exploratory analysis of PRIMA; (5) PARP inhibitors may be beneficial for stage IV ovarian cancer patients with inoperable disease and patients with prior neoadjuvant chemotherapy; and (6) there is an urgent need to increase awareness in both clinicians and patients on *BRCA* and HRD testing for optimizing treatment decision-making and improving clinical outcomes in newly diagnosed, advanced ovarian cancer patients. In clinical medicine, the limited availability of family history (FH) information and the complexity of FH criteria has hampered the implementation of BRCA testing. Moreover, many cancer patients with BRCA mutations are not tested because they do not meet the criteria for FH. Consequently, BRCA testing in many high income countries, including the US and Australia, is underused and used inappropriately, which has resulted in the loss of valuable opportunities for better cancer management and cancer prevention.

## Highlights

• Early *BRCA* testing is crucial and enables personalized treatment approach.
• *BRCA* mutation analysis is essential and should be utilized at the time of diagnosis.
• Single-agent PARP inhibitors seem most beneficial in *BRCA* mutations or HRD.
• Evidence suggests bevacizumab or niraparib-alone benefits for HRD-negative patients.
• HRD-tailored treatment should be explored further.

## 1 Introduction

### 1.1 Epidemiology, burden, and prognosis of advanced ovarian cancer

Ovarian cancer is the eighth most commonly occurring cancer and the seventh most common cause of cancer death in women globally (Ovarian Cancer Statistics: World Cancer Research Fund International, 2019, Ovary; GLOBOCAN, 2020). In 2020, it was estimated that there were 313,959 cases and 207,252 deaths due to ovarian cancer globally (Ovary; GLOBOCAN 2020). Though the overall incidence of cancer in low- and middle-income countries (LMICs) is lower compared to high-income countries (HICs), total cancer-related mortality rate is much higher in LMICs, more so for people under 65 years of age (Shah et al., 2019). In upper-middle income countries (UMIC) like Mexico, there is a huge challenge of a rapidly aging population. As a result, the relative survival rates of elderly cancer patients are significantly worse than those of younger individuals with an ever-widening gap between elderly and middle-aged cancer patients, which is thought to be due to poorer functional status, late diagnosis, and inadequate treatment (Aggarwal et al., 2015). Further, the actual burden (number of cases) is higher in LMICs because of population sizes (The World Ovarian Cancer Coalition Atlas, 2018). The GLOBOCAN study estimated that the highest number of incident cases of ovarian cancer in 2020 was in Asia with 170,759 cases, followed by 66,693 cases in the World Health Organization (WHO) European region, 26,630 cases in North America, 23,513 cases in Latin America and the Caribbean, 24,263 cases in Africa, and 2,101 cases in Oceania. The five-year prevalence estimates in these regions also follow a similar trend, with a reported 435,574, 190,105, 80,532, 62,165, 48,940, and 5,999 cases in the above regions, respectively. The number of ovarian cancer-associated deaths in Europe, Asia, Africa, Latin America and the Caribbean, North America, and Oceania in 2020 was 44,053, 112,936, 17,008, 15,266, 16,451, and 1,538 respectively (Ovary; GLOBOCAN 2020).

Delayed diagnosis may be one of the primary reasons for high ovarian cancer-associated mortality rates. Ovarian cancer is usually diagnosed at an advanced stage in 80% of women due to a lack of obvious symptoms during the early stage. At the time of diagnosis, cancer would have already spread throughout the abdominal cavity (Ovarian Cancer: American Cancer Society, 2018). According to the WHO, prevention, early detection and diagnosis, treatment and palliation are the key components of cancer control. However, these aspects are inadequately addressed in the LMICs which undermine the effective and sustainable cancer control in these regions. HICs are found to have better control of risk factors, education resources, increased screening and surveillance programs and improved cancer therapies, which results in decreased mortality rates compared to LMICs (Shah et al., 2019).

The high rates of delayed presentation and diagnosis of ovarian cancer may be attributed to various factors such as (The World Ovarian Cancer Coalition Atlas, Brain et al., 2014):

• Patient-related barriers such as low awareness of the disease and its symptoms, delay in seeking help, stigma surrounding cancer, and old age
• Healthcare-related barriers such as lack of adequate screening tools, access to diagnostics, trained clinicians, and referrals to specialist care
• Other country-specific factors (on a patient-to–patient basis)
• These barriers may collectively contribute to diagnostic delays and the increased numbers of patients presenting with advanced-stage ovarian cancer, leading to increased mortality (The World Ovarian Cancer Coalition Atlas, Brain et al., 2014). Stage I disease has approximately twice the five-year survival rate of stage IV disease (The World Ovarian Cancer Coalition Atlas). The overall five-year survival rate for ovarian cancer is 47%. However, the five-year survival rate of the majority of ovarian cancer patients (60%) who are diagnosed at stage IV is 29% (Ovarian Cancer: American Cancer Society). The 5-year cause-specific survival for serous carcinoma is 43%, whereas for endometrioid, mucinous, and clear cell carcinoma is 82%, 71%, and 66% respectively.

Survival also varies by race/ethnicity. For example, 5-year cause-specific survival for serous carcinoma is 36% in non-Hispanic black (NHB) women compared to 47%−48% in non-Hispanic white, Asian/Pacific Islanders (API) and Hispanic women. NHBs have the lowest survival rates across all stages of serous carcinoma, probably due to less adherence to guideline- based treatment. The relatively high survival in APIs for epithelial cancers overall reflects the low incidence of serous carcinoma, as well as high survival across subtypes (Torre et al., 2018).

### 1.2 Prevalence of *BRCA* mutations in advanced, high-grade, epithelial ovarian cancer

A high prevalence of germline *BRCA* (g*BRCA*) mutations (5.9%–29% *BRCA*1/2 in a sample size range of 14–141) has been reported in ovarian cancer cases (Alsop et al., 2012, Goncalves et al., 2019b, Gupta et al., 2021, Tyulyandina et al., 2019, Yoon, 2019, Goncalves et al., 2019a), particularly in advanced-stage, high-grade, epithelial ovarian cancer, in several studies from various regions across the globe (Alsop et al., 2012, Enomoto et al., 2019, Bu et al., 2019, Goncalves et al., 2019a, Goncalves et al., 2019b, Manchana et al., 2019, Kim et al., 2019, Ashour and Shafik, 2019, Song et al., 2014, Clinical Commissioning Policy, 2020) (Table 1). Therefore, early *BRCA* testing in epithelial ovarian cancer patients may help identify specific patient populations who may benefit from targeted therapies (Goncalves et al., 2019a, Goncalves et al., 2019b, Yoon, 2019).

**Table 1:** Prevalence of *BRCAm* in ovarian cancer patients across the globe.

	Overall *BRCA* prevalence	Prevalence of *BRCA*1	Prevalence of *BRCA*2	*BRCA* prevalence in high-grade serous ovarian cancer
Latin America^8^	29.8%^a^	–	–	–
Argentina^13^	32.4%^a^	–	–	–
UK^17,18^	15%^a^	3.8%^b^	4.2%^b^	11%^b^
Egypt^16^	21.15%^b^	68.2%^b^	–	25.7%^b^
Russia^6^	28.4%^a^	–	–	–
India^7^	25.5%^b^	–	–	–
Malaysia^10^	13.9%^b^	–	–	–
Thailand^14^	21.8%^b^	16.1%	5.7%	25.7%
South Korea^15^	–	–	–	39.8%^b^
China^12^	23.6%^b^	15.4% b	8.2% b	17.7% b
Japan^11^	14.7%^b^	9.9%^b^	4.7%^b^	28.5%^b^
Australia^9^	14.1%^b^	–	–	22.6%^b^

a Germline + somatic *BRCA* mutation.

b Germline *BRCA* mutations alone.

### 1.3 The current standard of care for first-line management of newly diagnosed, advanced ovarian cancer

For more than 20 years, platinum-based doublet chemotherapy (carboplatin and paclitaxel) has been used as the standard front-line chemotherapy for newly diagnosed, advanced ovarian cancer (du Bois, 2003, Ozols et al., 2003). The anti-VEGF monoclonal antibody, bevacizumab, was the first drug that conferred PFS benefits when given concurrently with first-line chemotherapy followed by maintenance treatment for approximately 12 months (du Bois, 2003, Ozols et al., 2003). Two key trials, the Gynecologic Oncology Group study (GOG-0218) (Burger et al., 2011) and the Gynecologic Cancer InterGroup (GCIG) International Collaboration on Ovarian Neoplasms (ICON7) (Perren et al., 2011), investigated the addition of bevacizumab concomitantly with standard chemotherapy and then as single-agent maintenance for the first-line treatment of ovarian cancer. On average, stage IV bevacizumab-concurrent plus maintenance patients had a median overall survival (OS) of 42.8 months as opposed to 32.6 months for stage IV control patients (HR, 0.75; 95% CI, 0.59 to 0.95). However, the ICON 7 trial did not show an OS benefit for bevacizumab. Both randomized, phase 3 trials show a significantly improved PFS with the addition of bevacizumab to standard chemotherapy in ovarian cancer patients at stage III–stage IV with poor prognosis (Table 2). Bevacizumab also exhibits survival benefits in patients with platinum-sensitive as well as platinum-resistant recurrent ovarian cancer (Aghajanian et al., 2012, Pujade-Lauraine et al., 2014).

**Table 2:** Summary of studies on the use of bevacizumab or PARP inhibitors for the management of newly diagnosed advanced ovarian cancer.

**First author [year] [study name]**	**Treatment arms [n]**	**Key endpoint outcomes**
Burger et al., 2011 [GOG-0218]	• Standard chemotherapy (1–6 cycles) + placebo (2–22 cycles) every 3 weeks [n = 625] • Standard chemotherapy (1–6 cycles) + bevacizumab (2–6 cycles) every 3 weeks + placebo (7–22 cycles) every 3 weeks [n = 625] • Standard chemotherapy (1–6 cycles) + bevacizumab (2–22 cycles) every 3 weeks (Dose – 15 mg/kg) [n = 623]	**Chemotherapy + bevacizumab throughout vs. chemotherapy + bevacizumab initiation vs. chemotherapy + placebo** **Median PFS:** 14.1 vs. 11.2 vs. 10.3 months; HR 0.908; 95% CI, 0.795 to 1.040; P = 0.16 and HR 0.717; 95% CI, 0.625 to 0.824; P < 0.001 **Median OS:** 39.7 vs. 38.7 vs. 39.3 months; p < 0.0095 HR 1.036; 95% CI, 0.827 to 1.297; P = 0.76 HR 0.915; 95% CI, 0.727 to 1.152; P = 0.45
Perren et al., 2011 [ICON7]	• Standard chemotherapy (1–6 cycles) every 3 weeks [n = 764] • Standard chemotherapy (1–6 cycles) + bevacizumab (1–5/6 cycles) every 3 weeks followed by bevacizumab maintenance for 12 additional cycles (Dose – 7.5 mg/kg) [n = 764]	**Standard-chemotherapy vs. Standard-chemotherapy + bevacizumab** **PFS:** 20.3 vs. 21.8 months; HR 0.81; 95% CI, 0.70 to 0.94; P = 0.004
Moore et al., 2018 [SOLO-1]	• Olaparib maintenance therapy after platinum-based chemotherapy (300 mg twice-daily) [n = 260] • Placebo [n = 131]	**Chemotherapy plus olaparib vs. placebo** **Rate of freedom from disease progression and from death at 3 years:** 60% vs. 27% HR 0.30; 95% CI, 0.23 to 0.41; P < 0.001 70% lower risk of disease progression with olaparib as compared to placebo
Ray Coquard et al. 2019 [PAOLA-1]	• Olaparib maintenance therapy after platinum-based chemotherapy (300 mg twice-daily) + Bevacizumab for 24 months [n = 537] • Placebo + bevacizumab maintenance for 24 months [n = 269]	**Olaparib + bevacizumab vs. placebo + bevacizumab** **Median PFS:** 22.1 vs. 16.6 months; HR 0.59; 95% CI, 0.49–0.72; P < 0.0001 **Median PFS by *tBRCAm* status:** ***tBRCAm*:** 37.2 vs. 21.7 months; HR 0.31; 95% CI, 0.20–0.47 **Non-*tBRCAm*:** 18.9 vs. 16.0 months; HR 0.71; 95% CI, 0.58–0.88 **Median PFS by HRD status:** **HRD positive, including *tBRCAm*:** 37.2 vs. 17.7 months; HR 0.33; 95% CI, 0.25–0.45 **HRD positive, excluding *tBRCAm*:** 28.1 vs. 16.6 months; HR 0.43; 95% CI, 0.28–0.66 **HRD negative/unknown:** 16.9 vs. 16.0 months; HR 0.92; 95% CI 0.72–1.17
González Martín et al., 2019 [PRIMA]	• Niraparib maintenance therapy after platinum-based chemotherapy (300 mg/200 mg once-daily) for 36 months [n = 484] (HRD, n = 245) • Placebo for 36 months [n = 244] (HRD n = 125)	**Niraparib vs. Placebo** **Median PFS in HRD population:** 21.9 vs. 10.4 months; HR 0.43; 95% CI, 0.31–0.59; P < 0.0001 **Median PFS in overall population:** 13.8 vs. 8.2 months; HR 0.62; 95% CI, 0.5–0.75; P < 0.0001
Coleman et al., 2019 [VELIA]	• Standard chemotherapy (1–6 cycles) + placebo followed by placebo maintenance (7–36 cycles) [n = 375] • Standard chemotherapy (1–6 cycles) + veliparib 150 mg twice daily followed by placebo maintenance (7–36 cycles) [n = 383] • Standard chemotherapy (1–6 cycles) + veliparib 150 mg twice daily followed by Veliparib 400 mg twice daily (7–36 cycles) [n = 382]	**Standard chemotherapy + Veliparib-throughout maintenance therapy vs. Standard chemotherapy + placebo** **Median PFS in *BRCAm* population:** 34.1 vs. 22.0 months; HR 0.44; 95% CI, 0.28–0.68; P < 0.001 **Median PFS in ITT Population:** 23.5 vs. 17.3 months; HR 0.68; 95% CI, 0.56–0.83; P < 0.001

PFS: Progression-free survival; HR: Hazard ratio; CI: Confidence interval; *BRCA*: Breast cancer mutations; HRD: Homologous recombination deficiency; PARP: Poly (adenosine diphosphate-ribose) polymerase; GOG: Gynecology Oncology Group.

Poly (ADP-ribose) polymerase (PARP) inhibitors facilitate single-strand DNA repair (Jiang et al., 2019, DiSilvestro and Secord, 2018). When PARP inhibitors bind to PARP at sites of single-stranded DNA breaks on replicating DNA strands, they trap the PARP. This in turn stalls replication forks; if they remain unresolved, they collapse and result in double-strand breaks. This can be resolved in cells with functional homologous recombination repair (HRR), whereby the double-strand breaks are successfully mended. However, in cells without functional HRR (such as those with *BRCA* mutations), erroneous repair results in increased DNA damage and cellular death (Gourley et al., 2019).

PARP inhibitors such as niraparib, olaparib, and rucaparib were approved in the US, Europe, Brazil, South Korea, and Australia by their respective drug regulatory agencies. Olaparib is approved for use, whereas niraparib is still in phase 2 trial in Japan. These three drugs have been shown to improve PFS, particularly in ovarian cancer patients with *BRCA* mutations (*BRCA*m) (Jiang et al., 2019, Moore et al., 2018, Pujade-Lauraine et al., 2017, González et al., 2019, Mirza et al., 2016, Mirza et al., 2016, Ray-Coquard et al., 2019). The landmark trials that have evaluated the efficacy and safety of PARP inhibitors in a first-line maintenance setting of advanced ovarian cancer include SOLO-1 (Moore et al., 2018), PAOLA-1 (Ray-Coquard et al., 2019), PRIMA (González Martín et al., 2019), and VELIA (Coleman et al., 2019) trials. Veliparib is still in the phase 3 trial stage in Brazil, Australia, South Korea, the US, UK, and Japan. Ongoing studies are evaluating the efficacy and safety of combining immune checkpoint inhibitors with PARP inhibitors. Some of these key trials include DUO-O/ENGOT OV-46 (NCT03737643: Clinical trials gov), KEYLYNK-001/ENGOT-ov43 (NCT03740165: Clinical trials gov), FIRST/ENGOT OV-44 (NCT03602859: Clinical trials gov), and ATHENA/GOG 3020/ENGOT OV-45 (NCT03522246: Clinical trials gov) trials.

Currently, BRCA mutation status/HRD testing are highly recommended to determine which maintenance treatment strategy (PARP inhibitor +/- Bevacizumab or Bevacizumab alone) should patient receive following standard first-line chemotherapy. (du Bois, 2003, Pujade-Lauraine et al., 2017).

Considering the increasing prevalence and high mortality rates of advanced ovarian cancer, and the growing importance of *BRCA* testing and the use of targeted therapies to improve treatment outcomes, this expert group meeting was convened to explore real-world practice patterns to distill the unmet needs and optimize diagnosis and management of newly diagnosed, advanced ovarian cancer.

## 2 Methods

A panel of 9 experts from LMIC (Egypt), UMIC (Argentina, Brazil, Colombia, Mexico and Russia) and HIC (Australia, Singapore and South Korea) (The World bank, 2021) convened in September 2019 to review current literature and key guidelines and share their real-world experiences on the management of newly diagnosed, advanced ovarian cancer. One external expert from UK was also a part of the panel to provide a global overview. The objectives of this meeting were to: (1) understand the burden and evolving landscape of first-line maintenance management of newly diagnosed, advanced ovarian cancer and (2) discuss the clinical implications of emerging data on optimization of genetic testing and selection and sequencing of first-line maintenance therapy of newly diagnosed, advanced ovarian cancer.

## 3 Incident cases of ovarian cancer in the participating regions

According to the GLOBOCAN 2020 data, ovarian cancer has been noted to be the eighth most common malignant tumor in women, with estimated incident cases of 23,513 cases per year in Latin America and the Caribbean (Ovary; GLOBOCAN 2020). The incidence and mortality rates of ovarian cancer in the participating countries from this region have been noted to be high with similar trends in estimates from the UK, Australia, Egypt, and other member countries in Asia (Table 3) (Ovary; GLOBOCAN, 2020, Ovary cancer: INCA, 2020, Ovarian Cancer Statistics: World Cancer Research Fund International, 2020).

**Table 3:** Prevalence and mortality rate of ovarian cancer in the participating regions (Ovary; GLOBOCAN 2020).

**Country**	**Prevalence of ovarian cancer (number of cases) (GLOBOCAN 2018)**	**Number of deaths from ovarian cancer (GLOBOCAN 2018)**
Brazil*	6,686	4,180
Argentina	2,330	1,321
Colombia	2,414	1,252
Mexico	4,579	2,765
UK	6,407	4,155
Egypt	2,674	1,934
Russia**	13,936	8,092
South Korea	2,656	1,225
Australia	1496	1002
Singapore	550	294

* As per the Brazilian National Cancer Institute (INCA), an estimated 6,650 new cases of ovarian cancer are reported to occur in Brazil, in 2020.^39^

** Russia had the thirteenth highest rate of ovarian cancer in 2018 with an age-standardized rate of 11.1 per 100,000 women.^40^

## 4 Evolving data for first-line maintenance therapy of newly diagnosed, advanced ovarian cancer

### 4.1 Guideline views

International guidelines recommend the following principles for the management of newly diagnosed, advanced ovarian cancer: (1) primary debulking surgery (PDS) in patients with good performance status where complete or optimal cytoreduction can be potentially achieved (Fotopoulou et al., 2017, ESGO-ESMO Consensus Conference on Ovarian Cancer, 2019, Armstrong et al., 2021); (2) systemic chemotherapy with three-weekly carboplatin/paclitaxel for six cycles remains the standard post-operative, first-line treatment in advanced stages after complete surgical staging (ESGO-ESMO Consensus Conference on Ovarian Cancer, Armstrong et al., 2021, Santaballa et al., 2016); (3) neoadjuvant chemotherapy with interval debulking surgery (IDS) after 3 cycles seems to be a good alternative. Both the EORTC and CHORUS trials showed the non-inferiority of the neoadjuvant chemotherapy approach, and a recent pooled study showed similar OS. (Vergote et al., 2011, Kehoe et al., 2015, Vergote et al., 2018); (4) bevacizumab should be included along with the initial chemotherapy in patients with macroscopic residual disease following standard surgery; bevacizumab can be continued during the maintenance period (ESGO-ESMO Consensus Conference on Ovarian Cancer; Armstrong et al., 2021, Santaballa et al., 2016); and (5) PARP inhibitors have shown greatest activity in patients with *BRCA*1/2 mutations (ESGO-ESMO Consensus Conference on Ovarian Cancer, 2019). Olaparib has been recommended as an optional agent for maintenance therapy in the NCCN guidelines for patients with stage II–stage IV disease and germline or somatic *BRCA*1/2 mutations with complete (CR) or partial remission (PR) following surgery and chemotherapy (Armstrong DK et al., 2021). However, evidence on the efficacy and safety of olaparib demonstrate benefit primarily for patients with stage III–stage IV disease.

### 4.2 Findings from recent landmark trials

The key clinical studies that assessed the efficacy and safety of PARP inhibitors in the management of newly diagnosed, advanced ovarian cancer include SOLO-1 and PAOLA-1 for olaparib, PRIMA for niraparib, and VELIA for veliparib (Table 2).

SOLO-1 was a double-blind, randomized, phase 3 trial that assessed the efficacy and safety of olaparib maintenance therapy (300 mg, twice daily) in patients with newly diagnosed, advanced FIGO stage III or IV high-grade serous or endometrioid ovarian cancer with a mutation in *BRCA*1, *BRCA*2 or both, who had a complete or partial clinical response after platinum-based chemotherapy (Moore et al., 2018, NCT01844986). Patients in this trial had either germline or a somatic *BRCA*1/2 mutation; hence, a substantial benefit was derived by this population from olaparib maintenance therapy after platinum-based chemotherapy. (Table 2) (Moore et al., 2018).

PAOLA-1 was a randomized, double-blind study that examined olaparib (300 mg, twice daily) with bevacizumab as maintenance therapy after first-line treatment in patients with newly diagnosed, advanced FIGO stage III, high-grade serous or endometrioid ovarian, primary peritoneal or fallopian tube cancer, regardless of *BRCA* status, who were in CR or PR to standard first-line platinum–taxane-based chemotherapy and bevacizumabThe greatest PFS benefit was observed in patients with *BRCAm* (37.2 vs. 21.7 months) and those with a positive homologous recombination deficiency (HRD) status including *tBRCAm* (37.2 vs. 17.7 months). (Ray-Coquard et al., 2019, ESMO Congress, 2019a, ESMO Congress, 2019b).

PRIMA was a double-blind, placebo-controlled phase III trial, which assessed the safety and efficacy of niraparib therapy in patients with newly diagnosed, advanced FIGO stage IIIB–IIIC, stage IV, high-grade serous or endometrioid ovarian, primary peritoneal, or fallopian tube cancer after response to first-line platinum-based chemotherapy, with a higher risk for recurrence and regardless of *tBRCAm*. The study findings revealed a clinically significant improvement in PFS with niraparib in the HRD-positive subgroup and overall population compared with placebo (HRD-positive subgroup: 21.9 vs. 10.4 months; p < 0.0001) (overall: 13.8 vs. 8.2 months; p < 0.0001) (Table 2).

The efficacy of veliparib vs. placebo was assessed in a phase III trial among patients with newly diagnosed, high-grade serous carcinoma, regardless of *tBRCAm*, HRD (using a lower cut-off value of 33), and NACT utilization. Patients were randomly assigned into three groups. (Mirza et al., 2016, NCT02470585). The patients receiving veliparib throughout experienced significantly extended PFS, compared to the control group, regardless of the biomarker, choice of surgery, or paclitaxel regimen. (Table 2) (Coleman et al., 2019).

Considering the evolving evidence on the efficacy of PARP inhibitors as first-line maintenance therapy in newly diagnosed, advanced ovarian cancer patients with *BRCA* and/or HRD, genetic testing to identify patients who are expected to derive maximum benefit from these agents may optimize the management of ovarian cancer in these patients. Currently, there is an urgent requirement for accessible, affordable, and standardized HRD testing methods across the world given the fact that these patients derive the most benefit from PARP inhibitors. The expert panel opined the need to optimize genetic and HRD testing as there is a lack of resources in most of the participating regions.

## 5 Optimization of genetic testing in newly diagnosed, advanced ovarian cancer

### 5.1 Key implications of emerging data on optimization of genetic testing

All the four key landmark trials, including PAOLA-1, SOLO-1, VELIA, and PRIMA, demonstrated a greater PFS benefit of PARP inhibitors in newly diagnosed, ovarian cancer patients with *BRCAm*; SOLO-1 is the only study that included 388 patients with centrally confirmed germline *BRCA*1/2 mutation and 2 patients with centrally confirmed somatic *BRCA*1/2 mutations (Moore et al., 2018, González et al., 2019, Ray-Coquard et al., 2019, NCT03737643: Clinical trials gov). The high frequency of *BRCAm* in ovarian cancer patients, as reported in several studies worldwide, warrants *BRCA* testing to identify the *BRCA* status in these patients. This may be done partly to guide therapy and partly to inform cascade testing and risk management in unaffected family members who carry germline mutations. The ESMO guidelines 2019 also recommend testing for *BRCA*1/2 mutations in advanced, non-mucinous ovarian cancer patients (Ovary cancer: INCA. 2020). Early screening for *BRCA*1 and *BRCA*2 mutations and initiation of genetic risk evaluation and *BRCA*1/2 testing following histological diagnosis have been recommended in the NCCN guidelines (Armstrong et al., 2021).

In countries with limited resources, there are no data on the cost-effectiveness of HRD genomic scar testing (Ngoi & Tan, 2021). Koldehoff et al. (2021) recently evaluated incremental cost-effectiveness ratios (ICERs) of targeted genetic testing for ovarian and breast cancers in countries such as Brazil, Canada, Germany, the UK, Australia, the US, Spain, and Norway. The following points were highlighted in the review:

• *BRCA* screening among high-risk women was associated with an ICER of $21,700/quality-adjusted life years (QALY).
• The ICERs for *BRCA* cascade screening (screening both affected and unaffected relatives) ranged between $6,500/QALY and $50,200/QALY.

According to the Markov model, population-based *BRCA* testing is considered cost-saving in high-income countries (HIC), and cost-effective in upper-middle–income countries (UMIC) from a societal perspective. On a similar note, *BRCA* testing is highly cost-effective in HIC and cost-effective in UMIC from a payer’s perspective. However, it is not cost-effective in LMIC from a societal as well as a payer’s perspective (Manchanda et al., 2020). Nevertheless, further research is required to evaluate or compare the role of genetic testing in improving diagnosis and treatment of ovarian cancer in LMICs.

Testing for *BRCAm* status is critical in predicting the treatment benefits of PARP inhibitors. Currently, *BRCA*1/2 genetic testing is suggested for ovarian cancer patients not only for estimating familial risk but also for determining their eligibility for PARP inhibitor therapy (Capoluongo et al., 2017). The panel also agreed that testing for *BRCAm* should be done as early as possible after the diagnosis of non-mucinous ovarian cancer to optimize treatment decisions, as long as resources are not constrained. Regarding the sequence of *BRCA* testing, some experts opined that tumor *BRCA* testing should be done first and if found positive, plasma germline testing should be done to confirm *gBRCA* mutations.

In addition to *BRCA* testing, evaluating the additional genes involved in the homologous recombination pathway is an evolving aspect of genetic testing to assist treatment choices. Guidelines have recommended considering testing for homologous recombination genes in ovarian cancer patients [41]. The presence of homologous recombination deficiency (HRD)-related gene mutations can be studied at the tumor DNA level by two tests that measure the level of scarring of three “*BRCA*mut-specific patterns” of genomic signatures, such as the Foundation Focus and the Myriad MyChoice®, which may predict the responsiveness of advanced ovarian cancer patients to PARP inhibitors (Capoluongo et al., 2017, Hoppe et al., 2019). Homologous recombination deficiency may be present in up to 50% of the patients with high-grade serous ovarian carcinomas (Heeke et al., 2018), but even patients who have tested negative for HRD have been shown to derive some benefit from PARP inhibitors maintenance (González et al., 2019, Coleman et al., 2017, Mirza et al., 2016;). In PAOLA-1, olaparib maintenance therapy along with bevacizumab has demonstrated a statistically and clinically significant PFS benefit in the ITT patient population beyond those with tumors harboring *BRCA* mutations, i.e. in *BRCA* non-mutant HRD-positive patients stratified as so by Myriad My Choice (Ray-Coquard et al., 2019). In the prespecified HRD subtype-wise exploratory analysis of the PRIMA population, niraparib appeared to have shown a PFS benefit in HRD/*BRCAm*-positive and *BRCAwt* patients who had tested negative for HRD. A significant difference in progression-free survival was observed between patients who received niraparib and those who had a placebo, irrespective of *gBRCA* mutations and HRD status (Mirza et al., 2016). In the HRD-negative AOC population, bevacizumab is currently available as maintenance therapy. Even though a synergy between PARP inhibitors and bevacizumab was expected, there was no clinical evidence supporting this in the subgroup of PAOLA-1 (Ray-Coquard et al., 2019) that tested for HRD-negative. The PRIMA trial (González Martín et al., 2019) showed that maintenance of niraparib alone improved PFS in patients with HRD-negative disease, suggesting it might be an alternative to bevacizumab in the absence of HRD. Nevertheless, the benefits of PARP inhibitor therapy are relatively modest compared to the risks. There is insufficient evidence for either of the trials, and further trials are required to determine which first-line maintenance therapy might be most effective for HRD-negative tumors.

During the meeting, although there was not an overwhelming consensus on the panel about the utility of HRD testing, the panel agreed to test for HRD, provided a reliable standardized affordable test is available in the country, even though they only provide a spectrum of degree of benefits with PARP inhibitors and do not account for PARP inhibitors resistance mechanisms. The panel also opined that the lack of an olaparib alone arm in PAOLA 1, and the inclusion of bevacizumab in the control arm might have contributed to the finding of no benefit in homologous recombination-proficient patients.

### 5.2 Proposed genetic testing considerations for newly diagnosed, advanced ovarian cancer

Beyond germline *BRCA*1/2 mutations, a small percentage of *BRCA*-related ovarian cancers harbor somatic mutations (Capoluongo et al., 2017). The concept of tumor testing has been introduced as an attractive approach that can potentially identify both germline and somatic *BRCA*1/2 pathogenic variants. Tumor testing can be used to identify an additional 3%–9% of patients with somatic *BRCA*1/2 mutations besides germline mutations and thus can save time and cost by identifying all the potential patients who can benefit from PARP inhibitor therapy. Subsequent genetic counseling and screening for germline mutations can then be recommended as necessary (Capoluongo et al., 2017).

The panel discussed the choice and order of *BRCA*1/2 sequencing in their respective countries, and most of the panel members mentioned that germline mutation sequencing is preferred for initiating diagnosis. Advisors also mentioned that some countries are currently looking into tumor testing first for diagnosis, and they expect more oncologists to take up this practice in the near future, particularly after tumor testing becomes reimbursable and a sufficiently low false negative and false positive rate is demonstrated.

Genetic testing may be beneficial for family members as enhanced screenings and prophylactic interventions can be performed. However, the risk of genetic discrimination and potential socio-economic implications of a positive result restricts the usage of germline genetic testing in several countries. Nevertheless, targeted germline and/or tumor *BRCA*1/2 mutation testing may be a potential alternative approach.

### 5.3 Current challenges in diagnostic testing for newly diagnosed, advanced ovarian cancer in the participating regions

There are several challenges associated with *BRCA* mutation testing. Interpretation of pathogenic mutations is often difficult, especially in the presence of variants of unknown significance. Furthermore, detection of somatic mutations and accurate determination of large genomic rearrangements from formalin-fixed, paraffin-embedded tumor samples is challenging (Capoluongo et al., 2017, Wallace, 2016). Although adoption of next-generation sequencing (NGS) technology may help in addressing these challenges and reduce processing time and overall unit costs, the complexity associated with NGS again confers a significant challenge (Wallace AJ 2016).

The expert panel discussed the current challenges in diagnostic testing for newly diagnosed, advanced ovarian cancer in the participating regions. The panel mentioned that the non-availability of an affordable and locally validated method of *tBRCA* sequencing or HRD tests was leading to variability in HRD test results between laboratories in countries such as Korea and Russia. In less-developed countries and remote areas, HRD testing appears to be less accessible. Although the HRD test is routinely advised (especially for high-grade serous ovarian cancer) in HIC such as in the UK, it is not a standard practice in all LMICs. The availability of tests and turnaround times may also vary between countries based on logistical arrangements and roll-outs specific to each country. In addition, the payer coverage policies and reimbursement issues also play a crucial role in HRD availability in countries. The panel also opined that there is a need to define optimal and validated Myriad MyChoice® “HRD scar” test results cut-off. Access to the HRD test (Myriad MyChoice®) is limited around the world, largely due to its out-of–pocket costs/lack of reimbursement. Additionally, the currently available HRD tests have limitations in terms of false-positive results since the presence of structural HRD changes may still be detected even when functional HRD no longer exists in the tumors after the development of PARP or platinum resistance (e.g. *BRCA* reversion mutations) (Mateo et al., 2019). There is also a possibility of false-negative results in terms of the ability of HRD tests to predict PARP inhibitor sensitivity. Evidence for this fact was provided in the translational analysis of PARP inhibitor studies involving relapsed ovarian cancer (Mirza et al., 2016, Coleman et al., 2017). HRD tests are available and reimbursed in developed nations such as the US. Recently, Myriad genetics received additional reimbursement in Japan. AmoyDx (China), Ambry Genetics (US), and SOPHiA Genetics (US) provide highly accurate HRD testing that goes beyond detecting HRR mutations.

The experts opined an important strategy to make HRD testing more available is to start with somatic tumor testing and triage further tests accordingly. Patients found to have HRD gene mutations on somatic testing can further be referred to genetics to identify if the mutation is somatic or germline. This strategy would facilitate rapid access to genomic information that can guide treatment options and lessen the burden on genetic counselors. Future studies must determine if the proposed strategy is viable based on the availability of testing resources and cost.

In clinical medicine, the limited availability of family history (FH) information and the complexity of FH criteria have hampered the implementation of BRCA testing. Moreover, many cancer patients with BRCA mutations are not tested because they do not meet the criteria for FH. Consequently, BRCA testing in many high income countries, including the US and Australia, is underused and used inappropriately, which has resulted in the loss of valuable opportunities for better cancer management and cancer prevention (Kemp et al. 2019).

## 6 Optimization of first-line maintenance therapy in newly diagnosed, advanced ovarian cancer

### 6.1 Key implications of emerging data on optimizing first-line maintenance therapy in newly diagnosed, advanced ovarian cáncer

Emerging clinical trials have established the role of combining bevacizumab and olaparib or niraparib for maintenance therapy after first-line chemotherapy among patients with newly diagnosed, advanced ovarian cancer. Clearly, HRD and *BRCAm* appear to derive the most benefit from this approach; however, the choice of treatment will need to be individualized based on tumor genetics, drug availability, individual drug tolerance, patients’ preferences, and socio-economic circumstances. Both *BRCA* and HRD scar testing or My Choice test should nonetheless be performed on all the patients to observe the degree of benefit from PARP inhibitors.

While niraparib or olaparib and bevacizumab may be preferred in stage III patients with visible residual disease after debulking surgery, all three PARP inhibitors (olaparib, niraparib, and veliparib) and bevacizumab may be beneficial for stage IV patients with the inoperable disease and patients with prior neoadjuvant chemotherapy and a *tBRCAmut* and *tBRCAwt* HRD-positive status (Burger et al., 2011, Perren et al., 2011, Moore et al., 2018, González et al., 2019, Ray-Coquard et al., 2019, Coleman et al., 2019). Furthermore, olaparib or niraparib has proven unequivocal PFS benefits in patients with germline or somatic *BRCA* mutations (Moore et al., 2018). In patients with advanced ovarian cancers that are *BRCAwt* but HRD-positive, olaparib plus bevacizumab or niraparib monotherapy may be the preferred choice (González et al., 2019, Ray-Coquard et al., 2019). Finally, bevacizumab or niraparib alone or observation (in case of CR to first-line chemotherapy) would be the current maintenance options for HRD-negative patients.

### 6.2 Applicability of evolving data to clinical practice settings in the participating regions

The panel agreed that the results of the PAOLA-1 and PRIMA studies should change the treatment paradigm in frontline *BRCAwt* ovarian cancer. In both studies, PARP inhibitors have demonstrated greater benefit in HRD-positive subgroup patients. However, the panel opined that it was uncertain if bevacizumab contributed to the PFS benefit in combination with olaparib given the lack of an Olaparib-alone arm. Furthermore, it is also important to consider the cost of the combination as not all countries have bevacizumab reimbursed or approved its use in the first-line setting.

Regarding the choice between niraparib and olaparib first-line maintenance, consideration needs to be given to the side-effect profile of niraparib (Table 4). Niraparib is a convenient option in terms of frequency (once per day), and toxicities are managed by dose reduction, especially in patients who weigh <77 kg in weight and/or have a baseline platelet count of < 150,000/µl. The panel members discussed the additional cost required for HRD scar testing to identify patients most suitable for niraparib and olaparib outside of *BRCA* mutation status. Ultimately, the results of PAOLA-1 and PRIMA will change practice only if the HRD tests and relevant drugs are broadly available and accessible. Another important factor to consider is that olaparib and bevacizumab are recommended for 24 months in PAOLA–1, whereas niraparib is recommended for 36 months in the PRIMA trial, and longer treatment duration may raise cost concerns, which may be counterbalanced by the cost of traveling and bevacizumab IV administration for the PAOLA-1 regimen. At present, there is no evidence of additional benefits from more prolonged therapy.

**Table 4:** Side-effect profiles of olaparib and niraparib.^59,60^

**Type of adverse reaction**	Common adverse reactions*	
	Olaparib, 2018	Niraparib, 2017
Blood and lymphatic disorders	Anemia	Thrombocytopenia Anemia Neutropenia Leukopenia
Gastrointestinal disorders	Nausea Vomiting Diarrhea Dyspepsia Constipation Stomatitis	Nausea Constipation Vomiting Abdominal pain/distention Mucositis/stomatitis Diarrhea Dyspepsia Dry mouth
Infections and infestations	Nasopharyngitis/upper respiratory tract infection/influenza	Urinary tract infection
General disorders and administration site conditions	Fatigue (including asthenia)	Fatigue/asthenia
Metabolism and nutrition disorders	Decreased appetite	Decreased appetite
Musculoskeletal and connective tissue disorder	Arthralgia/myalgia	Myalgia Back pain Arthralgia
Nervous system disorders	Dysgeusia Headache	Headache Dizziness Dysgeusia
Investigations	–	AST/ALT elevation
Cardiac disorders	–	Palpitations
Psychiatric disorders	–	Insomnia Anxiety
Respiratory, thoracic, and mediastinal disorders	–	Nasopharyngitis Dyspnea Cough
Skin and subcutaneous tissue disorders	–	Rash
Vascular disorders	–	Hypertension

* Includes all common, uncommon and very common side effects from clinical trials (>0.1%).

Due to the increasing use of PARP inhibitors as first-line treatment, it is important to carefully evaluate their effect on subsequent treatment efficacy. Clinical research in the next few years will focus on identifying short-term and long-term responders, and the mechanism of PARP inhibitor resistance. In ongoing and completed clinical trials, there remain unanswered questions related to timing and mode of treatment, the need for combining with antiangiogenic agents or immunotherapy and tailoring the treatment according to molecular subtype.

### 6.3 Expert choice of drugs for first-line maintenance therapy of advanced ovarian cancer

The panel discussed the changes in the treatment algorithm and guidelines for the management of advanced ovarian cancer. The experts stated their choice of PARP inhibitors with/without bevacizumab for first-line maintenance therapy in advanced ovarian cancer patients was based on the results of PAOLA-1, SOLO-1, and PRIMA studies, as shown in Table 5. Moreover, after first-line chemotherapy, olaparib maintenance treatment significantly extends PFS in patients with *BRCA*-mutation (Moore et al., 2018).

**Table 5:** Author’s Choice of PARP inhibitors and bevacizumab for first-line maintenance therapy of advanced ovarian cancer patients in the participating regions.

**Country**	***BRCAm***	***BRCAwt HRD+***	***BRCAwt HRD−***
Mexico	Olaparib based on SOLO1 results	Olaparib + bevacizumab or niraparib	Bevacizumab
Colombia	Olaparib based on SOLO1 results	Olaparib + bevacizumab or niraparib	Bevacizumab
Brazil	Olaparib	Olaparib + bevacizumab or niraparib	Bevacizumab
Australia	Olaparib based on SOLO-1 results	Olaparib + bevacizumab based on PAOLA-1 or niraparib depending on results of debulking surgery (olaparib and niraparib are not funded in Australia; there is no funding for HRD testing in Australia)	Bevacizumab (funded only for high-risk patients as per ICON-7)
Egypt	Olaparib	Niraparib (Influenced by cost)	Bevacizumab
Korea	Olaparib	Niraparib (need for standardization of HRD testing due to current variability of tissue HRD results)	Bevacizumab
Russia	Olaparib	Olaparib without bevacizumab	Bevacizumab (only for high-risk patients)
Argentina	Olaparib	Niraparib	Niraparib regardless of HRD status

HRD: Homologous recombination deficiency; *BRCAm:* BReastCAncer gene mutations; *BRCAwt:* BReastCAncer gene wild-type; PARP: Poly (adenosine diphosphate-ribose) polymerase.

## 7 Summary and future directives

The incidence of newly diagnosed, advanced ovarian cancer is increasing and is not expected to decline in the future, particularly in developing countries. Improved access to healthcare for expanding populations coupled with rapid urbanization and lifestyle changes may be some of the reasons for this rising incidence of ovarian cancer. Optimization of diagnosis through genetic testing and administration of timely and appropriate therapy may help improve the survival outcomes of this life-threatening disease. It is imperative to increase awareness and access to *BRCA* (both germline and somatic) and HRD testing to optimize patient outcomes. HRD testing can be useful for identifying patients who would benefit from PARP inhibitors therapy and also help understand the maintenance therapy options for patients after first-line platinum-based chemotherapy. However, the implementation of these strategies is limited by socioeconomic conditions and need to be improved.

## CRediT authorship contribution statement

**Jeffrey C.H. Goh:** Conceptualization, Data curation, Formal analysis, Funding acquisition, Investigation, Methodology, Project administration, Resources, Software, Supervision, Validation, Visualization, Writing – original draft, Writing – review & editing. **Charles Mclaren Gourley:** Conceptualization, Data curation, Formal analysis, Funding acquisition, Investigation, Methodology, Project administration, Resources, Software, Supervision, Validation, Visualization, Writing – original draft, Writing – review & editing. **David S.P. Tan:** Conceptualization, Data curation, Formal analysis, Funding acquisition, Investigation, Methodology, Project administration, Resources, Software, Supervision, Validation, Visualization, Writing – original draft, Writing – review & editing. **Angelica Nogueira-Rodrigues:** Conceptualization, Data curation, Formal analysis, Funding acquisition, Investigation, Methodology, Project administration, Resources, Software, Supervision, Validation, Visualization, Writing – original draft, Writing – review & editing. **Hesham Elghazaly:** Conceptualization, Data curation, Formal analysis, Funding acquisition, Investigation, Methodology, Project administration, Resources, Software, Supervision, Validation, Visualization, Writing – original draft, Writing – review & editing. **Marc Pierre:** Conceptualization, Data curation, Formal analysis, Funding acquisition, Investigation, Methodology, Project administration, Resources, Software, Supervision, Validation, Visualization, Writing – original draft, Writing – review & editing. **Gonzalo Giornelli:** Conceptualization, Data curation, Formal analysis, Funding acquisition, Investigation, Methodology, Project administration, Resources, Software, Supervision, Validation, Visualization, Writing – original draft, Writing – review & editing. **Byoung-Gie Kim:** Conceptualization, Data curation, Formal analysis, Funding acquisition, Investigation, Methodology, Project administration, Resources, Software, Supervision, Validation, Visualization, Writing – original draft, Writing – review & editing. **Flavia Morales–Vasquez:** Conceptualization, Data curation, Formal analysis, Funding acquisition, Investigation, Methodology, Project administration, Resources, Software, Supervision, Validation, Visualization, Writing – original draft, Writing – review & editing. **Alexandra Tyulyandina:** Conceptualization, Data curation, Formal analysis, Funding acquisition, Investigation, Methodology, Project administration, Resources, Software, Supervision, Validation, Visualization, Writing – original draft, Writing – review & editing.

## Declaration of Competing Interest

The authors declare the following financial interests/personal relationships which may be considered as potential competing interests: Jeffrey C.H. Goh – has been Advisory Board member, received sponsorship for conference and speaker’s bureau from AstraZeneca and Advisory Board Member for Tesaro. Charlie Gourley- has been part of advisory boards of Roche, Astrazeneca, MSD, Tesaro, Nucana, Clovis Foundation One, Sierra and has received research funding for Astrazeneca, Novartis, Aprea, Nucana and Tesaro. David SP Tan- has received research support from Astra Zeneca, Karyopharm Therapeutics, Bayer, Roche. Has been a part of Advisory Boards for Astra Zeneca, Roche, Bayer, MSD, Eisai, and Tessa Therapeutics and received honoraria from Astra Zeneca, Novartis, Roche, MSD, Bayer, Eisai, Takeda and Eisai. Angelica Nogueira-Rodrigues has been consultant for Eurofarma, Roche, Astra Zeneca, MSD, GSK and Eisai and received educational training from Roche, Eurofarma, Astra Zeneca, MSD. Hesham Elghazaly- No conflicts of interest. Marc Edy Pierre- No conflicts of interest. Gonzalo Giornelli- has been part of Adboard for AstraZeneca, Roche, MSD, Janssen Cilag and Speaker for AstraZeneca, MSD. Byoung-Gie Kim- No conflicts of interest. Flavia Morales – Vasquez- No conflicts of interest. Alexandra Tyulyandina- has received research support from Astra Zeneca, Roche, MSD and received honoraria from Astra Zeneca, Roche, MSD, Biocad, Tekeda, Pfiser, Eisai.
